# Myeloid malignancies with translocation t(4;12)(q11‐13;p13): molecular landscape, clonal hierarchy and clinical outcomes

**DOI:** 10.1111/jcmm.16895

**Published:** 2021-09-07

**Authors:** Vincent Parinet, Elise Chapiro, Audrey Bidet, Baptiste Gaillard, Odile Maarek, Laurence Simon, Christine Lefebvre, Sabine Defasque, Marie‐Joelle Mozziconacci, Anne Quinquenel, Matthieu Decamp, François Lifermann, Nadia Ali‐Ammar, Agathe Maillon, Marine Baron, Mélanie Martin, Stéphanie Struski, Dominique Penther, Jean‐Baptiste Micol, Nathalie Auger, Chrystèle Bilhou‐Nabera, Jean‐Alain Martignoles, Sylvie Tondeur, Florence Nguyen‐Khac, Pierre Hirsch, Damien Roos‐Weil, on behalf the FILO (French Innovative Leukemia Organization), GFCH (Groupe Francophone de Cytogénétique Hématologique) groups

**Affiliations:** ^1^ Sorbonne Université Service d'Hématologie Clinique Hôpital Pitié‐Salpêtrière APHP Paris France; ^2^ Sorbonne Université Unité de Cytogénétique Hôpital Pitié‐Salpêtrière APHP Paris France; ^3^ Centre de Recherche des Cordeliers Inserm Université de Paris Cell Death and Drug Resistance in Lymphoproliferative Disorders Team Sorbonne Université Paris France; ^4^ Laboratoire d’Hématologie Biologique CHU Bordeaux Bordeaux France; ^5^ Laboratoire d'Hématologie Hôpital Robert Debré Reims France; ^6^ Laboratoire de cytogénétique Centre Hospitalier de Troyes Troyes France; ^7^ Hematology Laboratory Hôpital Saint‐Louis APHP University of Paris Paris France; ^8^ Laboratoire de Génétique des Hémopathies CHU Grenoble Alpes Grenoble France; ^9^ Secteur cytogénétique hématologique Laboratoire CERBA Saint‐Ouen l'Aumône France; ^10^ Laboratoire de cytogénétique et biologie moléculaire Institut Paoli‐Calmettes Marseille France; ^11^ CHU de Reims Hôpital Robert Debré Reims France; ^12^ Unité de Formation et de recherche (UFR) Médecine Université Reims Champagne‐Ardenne Reims France; ^13^ Service de Génétique CHU de Caen Normandie Caen France; ^14^ Department of Internal Medicine Centre Hospitalier de Dax Dax France; ^15^ Laboratoire de Cytogénétique CHU Caremeau Nîmes France; ^16^ Laboratoire d’hématologie/Plateau Technique Hématologie‐Oncologie IUCT Oncopole Toulouse France; ^17^ Laboratoire de Génétique Oncologique CLCC Henri Becquerel & INSERM U1245 Rouen France; ^18^ Hematology Department Gustave Roussy Paris‐Saclay University Villejuif France; ^19^ Laboratoire de Cytogénétique Institut Gustave Roussy Villejuif France; ^20^ Service d’Hématologie Biologique Unité de Cytogénétique onco‐hématologique Hôpital Saint‐Antoine APHP Sorbonne Université Paris France; ^21^ Département d’hématologie biologique INSERM Centre de Recherche Saint‐Antoine Sorbonne Université, AP‐HP Hôpital Saint‐Antoine Paris France

**Keywords:** acute myeloid leukaemia, CHIC2, ETV6, myelodysplastic syndrome, prognosis, t(4;12)

## Abstract

Translocation t(4;12)(q11‐13;p13) is a recurrent but very rare chromosomal aberration in acute myeloid leukaemia (AML) resulting in the non‐constant expression of a *CHIC2*/*ETV6* fusion transcript. We report clinico‐biological features, molecular characteristics and outcomes of 21 cases of t(4;12) including 19 AML and two myelodysplastic syndromes (MDS). Median age at the time of t(4;12) was 78 years (range, 56–88). Multilineage dysplasia was described in 10 of 19 (53%) AML cases and CD7 and/or CD56 expression in 90%. FISH analyses identified *ETV6* and *CHIC2* region rearrangements in respectively 18 of 18 and 15 of 17 studied cases. The t(4;12) was the sole cytogenetic abnormality in 48% of cases. The most frequent associated mutated genes were *ASXL1* (n = 8/16, 50%), *IDH1*/*2* (n = 7/16, 44%), *SRSF2* (n = 5/16, 31%) and *RUNX1* (n = 4/16, 25%). Interestingly, concurrent FISH and molecular analyses showed that t(4;12) can be, but not always, a founding oncogenic event. Median OS was 7.8 months for the entire cohort. In the 16 of 21 patients (76%) who received antitumoral treatment, overall response and first complete remission rates were 37% and 31%, respectively. Median progression‐free survival in responders was 13.7 months. Finally, t(4;12) cases harboured many characteristics of AML with myelodysplasia‐related changes (multilineage dysplasia, MDS‐related cytogenetic abnormalities, frequent *ASXL1* mutations) and a poor prognosis.

## 1 INTRODUCTION

Acute myeloid leukaemia (AML) is the most common form of acute leukaemia, with an incidence that increases with advanced age. This malignant disorder emerges from a transformed haematopoietic stem/progenitor cell (HSPC) that acquires multiple genomic and chromosomal alterations, ultimately evolving into clinically overt disease. Molecular and cytogenetic abnormalities cooperate by successive steps to initiate AML and involve critical genes of normal cell development, survival, proliferation and maturation.^1^, ^2^ Identified aberrations in AML have known or putative consequences on different cell functions including signalling pathways, chromatin modification, DNA methylation, transcription factors, splicing machinery and tumour suppressors.^1^

Cytogenetic analysis constitutes a mandatory part of AML diagnostic workup through identification of chromosomal abnormalities such as translocations, inversions or gain/deletions. It identifies biologically distinct subsets of AML that differ in their response to therapy and treatment outcome, and thus plays a major role in risk stratification.^3^ Cytogenetic analyses of large cohorts of AML patients have shown that translocations/inversions generating chimeric fusion genes underlie disease pathogenesis in around 50% and 30% of AML arising in children and younger adults, whereas in older adults only a minority of AML have balanced rearrangements.^4^ Main translocations in AML are represented by translocation t(15;17)(q24;q21) (10–15%), and translocations involving core‐binding factor (CBF) (*RUNX1*‐*RUNX1T1*, *CBFB*‐*MYH11*) (10–15%) and *KMT2A* (4–5%).^4^, ^5^ When these events occur in HSPC, they are considered critical initiating steps in the pathogenesis of AML, as they first appear in the clonal history,^6^, ^7^ or can lead to pre‐leukaemic haematopoietic in xenotransplantation models.^5^, ^8^

Translocation (4;12)(q11‐13;p13) [t(4;12)] is a recurrent but extremely rare AML cytogenetic abnormality with an estimated incidence below 1%.^9^, ^10^ It results in the non‐constant expression of a *CHIC2*/*ETV6* fusion transcript.^11^, ^12^ The 12p13 breakpoint invariably involved the *ETV6* gene, while 4q11‐12 breakpoints fell in a genomic region centromeric to *GSX2*, also including *CHIC2*, *FIP1L1* and *PDGFRA*.^11^, ^12^, ^13^, ^14^, ^15^ All these four latter genes have been described as partner genes to *ETV6*. There have been about forty reported cases in literature over the last 25 years through scattered case reports and small series.^14^, ^15^, ^16^ These previous reports highlighted certain specific clinical and biological features and a likely unfavourable prognosis with a median survival of less than one year.^14^, ^15^, ^16^ Main reported biological characteristics included the presence of trilineage dysplasia, and frequent CD7 expression along with low or absent myeloperoxidase (MPO) activity.^14^, ^15^ In contrast, limited data are available regarding molecular characteristics and possible cooperating mutations to t(4;12).^15^

The main objectives of this national multicentric retrospective study were (i) to identify cases of t(4;12)(q11‐13;p13) malignancies, (ii) to describe their cytological, immunophenotypic, cytogenetic and molecular characteristics, as well as clinical outcomes, and (iii) to address clonal architecture of this entity, while studying the largest patient cohort to date.

## 2 PATIENTS, MATERIAL AND METHODS

### 2.1 Patients

This national multicentric retrospective and observational study includes 21 patients with a t(4;12)(q11‐13;p13) diagnosed between 1995 and 2020. Identification of t(4;12)(q11‐13;p13) by conventional cytogenetic analysis was the unique inclusion criteria. Written consent to blood collection and biological analyses were obtained in accordance with the Declaration of Helsinki and its later amendments and with ethical approval from ethics committee (CNIL 2212382).

Diagnoses of myeloid neoplasms were based on morphologic, immunophenotypic, cytogenetic and molecular criteria defined by the WHO 2016 classification.^17^ Clinical and standard biological features were recorded at diagnosis of myeloid neoplasms or at the time of t(4;12) identification. Multilineage dysplasia was defined according to the WHO 2016 classification. Type of therapy was categorized as supportive therapy, low‐intensity chemotherapy (low‐dose cytarabine or other cytotoxic agents), hypomethylating agents (5‐azacitidine or decitabine), induction chemotherapy and allogeneic haematopoietic stem cell transplantation (SCT). Data regarding immunophenotypic analysis by flow cytometry were available in 15 of 19 AML cases. Flow cytometry was performed at each institution using comprehensive acute leukaemic panels in accordance with their protocols. Panels commonly included CD11b, CD13, CD33, CD34, CD45, CD64, CD117, myeloperoxidase (MPO), surface and cytoplasmic CD3, CD4, CD5, CD7, CD8, CD19, CD20, HLA‐DR and terminal deoxynucleotidyl transferase (TdT). French flow cytometric centres apply the consensus proposal of the European LeukemiaNet.^18^ Briefly, the leukaemic cells were identified using a CD45 vs. SSC strategy (dim expression of CD45 with low SSC properties) and positivity for a given marker was defined either by (i) the use of a threshold of 10% or 20% of positive cells (depending on the date of diagnosis and related recommendations) or (ii) the assessment of the fluorescence shift and pattern of the whole clonal leukaemic population, by comparison with fluorescence intensity of the tested marker on negative cells in the same sample.

### 2.2 Conventional cytogenetic and FISH analyses

Chromosome banding (CB) analyses were performed according to the usual techniques to obtain G‐ or R‐banded chromosomes from BM or PB samples. All karyotypes were described according to the International System for Human Cytogenetic Nomenclature (ISCN 2020). Complex karyotypes were defined as the presence of three or more numerical or structural chromosomal abnormalities. FISH was performed on interphase nuclei and metaphases, following standard procedures and using specific probes: FIP1L1/CHIC2/PDGFRA (4q12) tricolour, Vysis (Abbott Molecular, Des Plaines, IL); FIP1L1/CHIC2/PDGFRA (4q12) deletion/fusion (Cytocell, Cambridge, UK); and ETV6 (12p13) break‐apart dual colour, Vysis (Abbott) (see Figure S1).

### 2.3 Molecular and clonal architecture analyses

DNA was extracted from BM aspirates at the time of t(4;12) identification. Mutations were identified using Sanger sequencing and/or targeted next‐generation sequencing (NGS) as previously described with minor modifications and using a panel of 42 genes mutated in AML and myeloid malignancies (Table S1).^19^ Clonal architecture of t(4;12) samples was reconstructed by combining cytogenetic and molecular data (Tables S2 and S3). The order of lesions was inferred from mutational VAF and chromosomal abnormality frequencies as previously described^7^ and was defined as clonal/initiating or subclonal/secondary events. Briefly, to determine the order of chromosomal and genomic lesions in each AML sample, we first determined variant allele frequencies (VAFs) using the following quantitative results: (1) frequencies of interphasic nuclei (in 8/9 samples) with t(4;12) translocation, deletions or gains, or metaphases if interphase FISH not available (1/9), (2) VAFs from targeted sequencing runs for somatic gene mutations (single nucleotide variants and indels), and (3) VAFs from targeted sequencing for SNPs in sequenced regions with losses of heterozygosity (LOHs) or copy‐number variations. These quantitative values were converted to fractions of cells harbouring the lesions (variant cell fraction, VCF), taking into account LOHs, with or without copy‐number variations, as well as the gender of the patient for lesions on chromosome X. Then, 95% confidence intervals (CI) were calculated by using the numbers of analysed nuclei and/or read depths as sample sizes for cytogenetic and NGS quantifications, respectively. The formula used to define 95% CI was the following one:

$$\text{IC(95) =}\;\left[p‐1,96\sqrt{\frac{p(1‐p)}{n}};p+1,96\sqrt{\frac{p(1‐p)}{n}}\right]$$

*(p *= variant cell fraction; *n *= number of reads [NGS] or analysed nuclei [interphase FISH]).

VCFs were considered as different if their 95% CI (lower and upper limits) did not overlap. All data regarding VAFs, VCFs and 95% CI for each sample represented in Figure 2 are now provided in Table S5.

### 2.4 Outcome analysis and statistics

Response to treatment followed standard international criteria.^20^ The Kaplan‐Meier analyses were performed to construct survival curves, and the log‐rank test was used to determine differences between groups using the IBM SPSS Statistics software for Windows (version 25.0; IBM, Armonk, NY). The chi‐square test was used to compare data distribution in different cytogenetic subgroups. The threshold for statistical significance was set to *p *< 0.05.

## 3 RESULTS

### 3.1 Patients and disease characteristics

We identified 21 cases harbouring a t(4;12)(q11‐13;p13) including 19 AML and 2 myelodysplastic syndromes (MDS). Main clinical and biological characteristics of the cohort are summarized in Table 1. The male/female ratio was 2/1. Median age of the cohort at the time of t(4;12) diagnosis was 78 years (range, 56–92). Median WBC level in AML cases was 18.2 × 10^9^/L (range, 0.7–82.9). Three AML cases were secondary (two post‐MPN [one primary myelofibrosis and one atypical CML] and one therapy‐related [anthracycline‐based regimen for diffuse large B‐cell lymphoma]). Multilineage dysplasia was described in 10 of 19 AML cases (53%). Immunophenotypic analysis of AML blasts by flow cytometry showed constant CD33 expression in the 15 tested cases, including strong expression in 12 cases. CD34 and CD117 were expressed in nearly all cases (14/15, 93%). MPO expression was positive in only 23% of cases. CD7, CD56 and CD4 aberrant expressions were found in respectively 79%, 63% and 36% of cases (Table 1). All tested cases were negative for CD3, CD5, CD10, CD19, CD20 and TdT.

**TABLE 1:** Main clinical and biological characteristics of t(4;12)(q11‐13;p13) cohort

Characteristics	n (%)
Total	21
Median age, years (range)	78 (56–92)
Gender, male/female	14 (67)/7 (33)
Median WBC, 10^9^/L (range)	18.2 (0.7–82.9)
Myeloid malignancies	
MDS with excess blasts^a^	2/21 (10)
AML (FAB classification)	19/21 (90)
AML 0	5/19 (26)
AML 1	5/19 (26)
AML 2	5/19 (26)
AML 4	1/19 (5)
Unspecified	3/19 (15)
AML (WHO classification)	19/21 (90)
AML with recurrent genetic abnormalities	
AML with mutated *NPM1*	1/19 (5)
AML with mutated *RUNX1* ^b^	2/16 (25)
AML with myelodysplasia‐related changes	12/19 (63)
Therapy‐related AML	1/19 (5)
AML, NOS	3/19 (5)
Cytological characteristics of AML	
Multilineage dysplasia	10/19 (53)
Flow cytometry findings of AML blasts	
CD33+	15/15 (100)
CD7+	11/14 (79)
CD56+	7/11 (63)
CD7+ and/or CD56 +	13/14 (93)
MPO+	3/13 (23)
Conventional karyotype	
Isolated t(4;12)	10/21 (48)
One additional CA	7/21 (33)
Complex karyotype	4/21 (19)
FISH	
*ETV6* rearrangement	18/18 (100)
*CHIC2* region rearrangement	15/17 (88)

Abbreviations: CA, chromosomal abnormality; MDS, myelodysplastic syndrome; MPN, myeloproliferative neoplasm; NOS, not otherwise specified; WBC, white blood cell count.

^a^ One MDS with excess blasts (EB)‐1 (7%) and one with EB‐2 (11%).

^b^ Two other cases with *RUNX1* mutations were included in AML with myelodysplasia‐related changes.

### 3.2 Cytogenetic features

**FIGURE 1:**
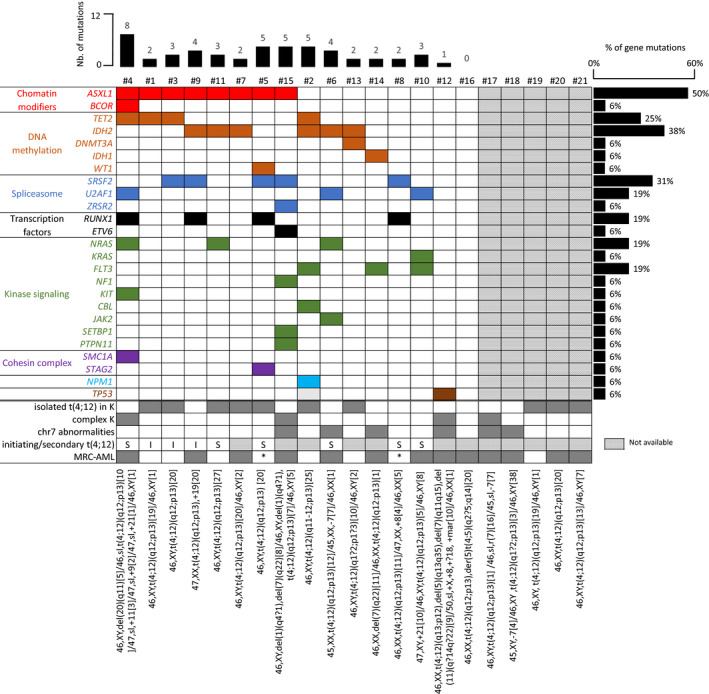
Mutational and cytogenetic analyses of t(4;12) patients. Targeted sequencing data were available for 16 of 21 patients (complete list of targeted genes and mutations available in Tables S1 and S3). Each column represents a patient sample and each row a mutated gene. Mutated genes have been grouped by different functional categories. The percentage of each mutated gene in the whole cohort is indicated on the right of the grid, the number (nb) of mutations per sample on the top, and the complete karyotype by ISCN at the bottom. Coloured and dark grey box: presence; white box: absence; hatched boxes: not available. Abbreviations: I, initiating; S, secondary; K, karyotype, MRC‐AML, myelodysplastic‐related changes‐AML; NA, not available; *, not applicable (MDS with excess blasts)

The translocation t(4;12)(q11‐13;p13) was detected at diagnosis in 18 of 21 cases (86%) and at relapse in 3 of 21 cases (14%). In these three cases, the initial karyotype was available and did not find the t(4;12). FISH analyses highlighted rearrangements of *ETV6* in 18 of 18 cases (100%) and of *CHIC2* region in 15 of 17 (88%) cases. The t(4;12) was the sole identified cytogenetic abnormality in 48% of cases (n = 10, Table 1 and Figure 1). The number of chromosomal aberrations ranged from 1 to 8. Monosomal and complex karyotypes were observed in respectively 2 of 21 (10%) and 4 of 21 (19%) cases. Considering additional cytogenetic abnormalities present in 11 patients, abnormalities of chromosome 7 were the most frequent (n = 6/11, 55%; including three complete loss and three 7q deletions (del), followed by del5q (n = 2, 18%), trisomy 8 (n = 2, 18%) and trisomy 21 (n = 2, 18%)) (Figure 1 and Table S2). Among these 11 cases, the t(4;12) was in an initiating (n = 3), secondary (n = 3) or independent clone (n = 5). No del17p was identified.

### 3.3 Molecular and clonal architecture analyses

*FLT3*‐ITD and *NPM1* mutational statuses were available in all 19 AML cases and were respectively present in three (16%) and one (5%) patients. Targeted NGS was performed in 16 cases. A total number of 56 mutations in 41 genes were found with a median number per patient of three (range, 0–10). The most frequently associated mutated genes were *ASXL1* (n = 8/16, 50%), *IDH2* (n = 6/16, 38%), *SRSF2* (n = 5/16, 31%), *TET2* (n = 4/16, 25%) and *RUNX1* (n = 4/16, 25%) (Figure 1 and Tables S3 and S4). Considering functional categories of mutated genes, mutations in genes implicated in DNA methylation (*DNMT3A*, *TET2*, *IDH1*, *IDH2*), chromatin modifier (*ASXL1*, *BCOR*), splicing (*ZRSR2*, *SRSF2*, *U2AF1*) and signalling pathways (*FLT3*, *NRAS*, *KRAS*, *NF1*, *KIT*, *CBL*, *JAK2*) were respectively present in 68%, 47%, 47% and 58% of screened samples (Table S6).

**FIGURE 2:**
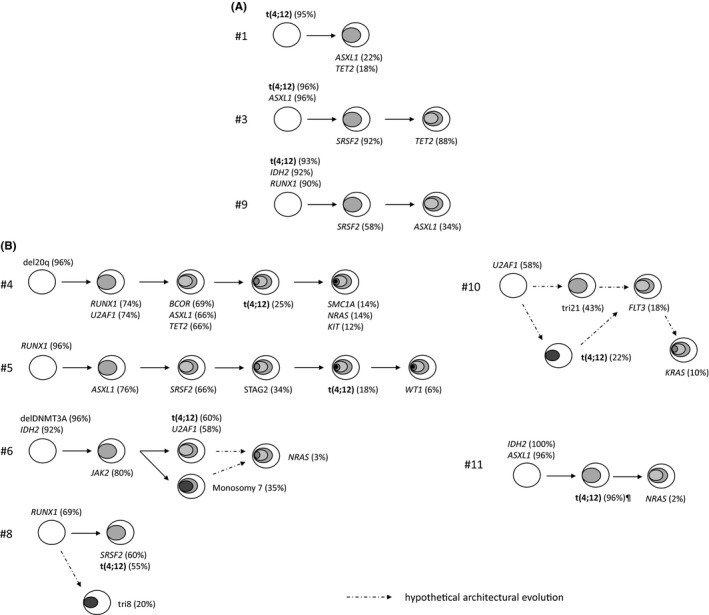
Clonal architecture of t(4;12) samples. Panels A and B respectively represent initiating and secondary t(4;12). The order of cytogenetic and mutational events was inferred from variant cell fractions (VCF) of identified mutations and cytogenetic abnormalities frequencies (Table S5), as previously described.^7^ This analysis only included analysis of genomic lesions at the time of t(4;12) identification and did not represent follow‐up samples of the same patient. The precise clonal architecture was difficult to infer in samples #6 and #10 because chromosomal abnormalities were identified in independent clones by conventional karyotype: t(4;12) and monosomy 7 for sample #6; and t(4;12) and trisomy 21 for sample #10. This is thus not possible to infer if subclonal mutations (ie NRAS in #6 or FLT3 in #10) belong to the t(4;12) clone or another clone (monosomy 7 in #6, trisomy 21 in #10). **¶** Subclonality of t(4;12) in case #11 was inferred thanks to analysis of relapse sample in which the t(4;12) detected by interphase FISH was present in 2%, while VAFs of *IDH2* and *ASXL1* mutations were respectively 28 and 24%

By combining cytogenetic and molecular data, we then attempted to reconstruct the clonal architecture of t(4;12) samples. Data were conclusive for nine cases represented in Figure 2. As previously described in myeloid malignancies,^7^, ^21^ the first/early events were mainly mutations in epigenetic regulators (*ASXL1*, *DNMT3A*, *IDH2*; cases #3, #6, #9, #11), chromosome 20q deletions (case #4), transcription factors (*RUNX1*; cases #5, #8) or splicing machinery (*U2AF1*, case #10), with a frequent early accumulation of epigenetic events in a dominant clone. These early events were recurrently followed by lesions affecting the splicing machinery (*U2AF1*, *SRSF2*; cases #3, #4, #5, #6, #8), transcription factors (*RUNX1*; case #4) or cohesin complex (*SMC1A*, *STAG2*; cases #4, #5) and then mutations in signalling pathways (*NRAS*, *KRAS*, *FLT3*, *KIT*; cases #4, #6, #10, #11) **(**Figure 2). Interestingly, t(4;12) events could be either an initiating (n = 3) (Figure 2A, including one case [#1] in which t(4;12) was the sole first hit) or secondary (n = 6) (Figure 2B, including two cases [#4, #5] in which t(4;12) represented one of the latest events) lesion. In the 3 cases in which t(4;12) was an initiating event, it was not associated with mutations in signalling pathways contrary to what was observed when it was a secondary event (Figure 2 and Table S6bis).

### 3.4 Outcomes

Among the whole cohort, five (24%) patients only received best supportive care (n = 3) or hydroxycarbamide (n = 2). The 16 (76%) others patients received antitumoral treatment (Table 2), which consisted of induction chemotherapy (anthracycline‐cytarabine–based regimen; n = 5/16, 31%), hypomethylating agents (n = 5/16 including one MDS, 31%), imatinib (n = 2/16, 13%), IDH1/2 inhibitors (n = 2/16, 13%), FLT3 inhibitor (n = 1/16, 6%) and venetoclax (n = 1/16, 6%).

**TABLE 2:** Treatment and outcomes of t(4;12) patients

	n (%)	
Total	21	
Overall survival		
Median (95% CI), months	7.8 (3–23)	
Antitumoral therapies	16/21 (76)	
Induction therapy	5/16 (31)	
Hypomethylating agents	5/16 (31)	
Others	6/16 (38)	
Response		
ORR	6/16 (38)	
PR	1/16 (7)	
CR	5/16 (31)	
Induction chemotherapy		
ORR	2/5 (40)	
CR	2/5 (40)	
Refractory	3/5 (60)	
Hypomethylating agents		
ORR	4/5 (80)	
PR	3/5 (60)	
CR	1/5 (20)	
Refractory	1/5 (20)	
Others		
ORR	0/6	

Abbreviations: CI, confidence interval; CR, complete remission; ORR, overall response rate; PR, partial response.

Among these 16 patients, overall response rate (ORR) was 37% (n = 6) including 31% CR (n = 5) and 6% PR (n = 1). ORR with induction chemotherapy and hypomethylating agents were respectively 40% (n = 2/5, two CR) and 80% (n = 4/5, three CR and one PR). No response was observed with other regimens. The median number of total therapeutic lines was 1 (range, 0–2). Only one patient received allogeneic SCT (Table 2).

**FIGURE 3:**
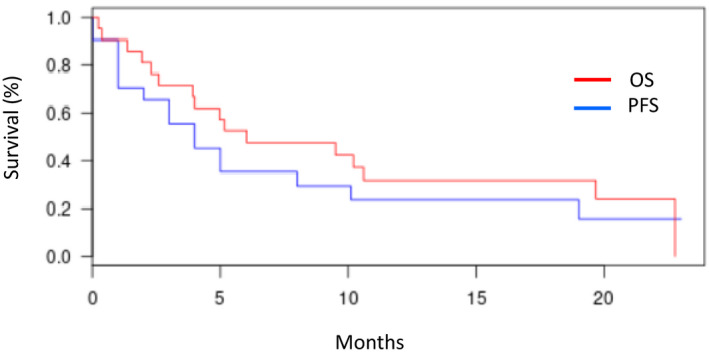
Overall survival (OS) (red) and progression‐free survival (PFS) (blue) for the whole cohort of t(4;12) patients

Median PFS and OS (95% CI) for the entire cohort were respectively 5 (2–20) and 7.8 months (3–23) (Figure 3). At last follow‐up, only 5 of 21 (23.8%) patients were alive. Median OS was 19.7 months for patients in CR after first‐line therapy, 19 months for patients who received induction chemotherapy and 11 months for those treated with hypomethylating agents (*p *= 0.8 between induction chemotherapy and hypomethylating agents). Deaths (n = 16) were related to AML progression (n = 12), infectious complications (n = 3) and unknown cause (n = 1).

## 4 DISCUSSION

To our knowledge, this is the largest retrospective series of t(4;12) haematological malignancies available to date with 21 cases, while so far only about forty have been published. In this series, we not only confirmed many of the already published clinical and biological features but also extended our knowledge of particular characteristics and highlighted molecular and clonal architecture of this rare entity. Most cases were AML with multilineage dysplasia, but we also identified two cases of MDS with excess blasts, which had not been described before. We confirmed the frequent expression of CD7 as seen in other AML series along with rare expression of MPO. In contrast to the study of Li and al.^15^ in which CD56 was expressed in only 1 of 14 cases, CD56 expression was frequent in our cohort (n = 7/11, 63%).

Chromosomal rearrangements generating oncologic fusion proteins are considered critical initiating events that usually play a central role in leukaemogenesis. Breakpoints in t(4;12) invariably involved *ETV6* for 12p13 and fell in a genomic region centromeric to *GSX2* and frequently involved *CHIC2* and *PDGFRA* for 4q11‐q12.^11^, ^12^, ^15^ Our FISH data confirmed these breakpoints in almost all cases. Translocation involving the 12p13 band with *ETV6* is one of the most frequently described chromosomal abnormality in MDS and AML.^22^ *ETV6* codes a ubiquitous nuclear protein that belongs to the ETS (E‐twenty‐six) family of transcription factors and plays a central role in embryogenesis and haematopoietic regulation. Different partner genes to *ETV6* have been described in t(4;12) including *CHIC2*, *GSX2* and *PDGFRA*. The impact of *CHIC2*/*ETV6* fusion transcript in t(4;12) leukaemogenesis has remained controversial as its expression was inconstant. *CHIC2* codes for a CHIC (cysteine‐rich hydrophobic domain) family protein, involved in plasma membrane and vesicular function,^23^ which precise function in haematopoietic is not yet known. A role for PDGFRA has also been suggested. PDGFRA is a member of the class III receptor tyrosine kinase family that activates intracellular signalling pathway by forming homodimer or heterodimer with PDGFRB.^24^ Considering the possible implication of PDGFR signalling in t(4;12) pathogenesis, several teams tried to use kinase inhibitors, which resulted in variable success.^15^ Imatinib was used in two cases of our cohort but did not lead to any objective response. *GSX2* (GS homeobox 2), coding for a transcription factor usually expressed in embryogenesis and in the adult central nervous system, is also a putative oncogene as *GSX2* was overexpressed in t(4;12) cases and had transforming potential in a fibroblastic cell line.^12^, ^13^ Several hypotheses have been raised regarding the impact of t(4;12) on AML pathogenesis: i) addition of deregulating enhancer elements near *ETV6* region; ii) disruption of normal DNA binding function of ETV6 although keeping intact its homodimerization; and iii) interference or abolition of PRC2 binding sites near *GSX2* locus leading to GSX2 overexpression. Further studies using RNA sequencing and/or chromatin immunoprecipitation along with gene expression profile may respectively help describe more precisely t(4;12) breakpoints and provide a better understanding of the CHIC2 or PDGFRA/ETV6 fusion protein and/or GSX2 role in t(4;12) myeloid malignancies.

Very limited molecular data were available in t(4;12) cases,^15^ identifying *FLT3*‐ITD, *IDH2* and *JAK2* mutations in respectively 2/11, 1/2 and 1/2 studied cases. No mutation in other genes was described, but the number of samples screened was small (*KRAS*/*NRAS*, n = 8; *CEBPA*/*KIT*/*NPM1*, n = 3; *IDH1*, n = 2). Our more comprehensive approach identified mutations in several genes unprecedently described in this particular AML. We found frequent implication of genes involved in DNA methylation (*TET2*, *IDH1*, *IDH2*), chromatin modifier (*ASXL1*), transcription factors (*RUNX1*) and splicing machinery (*SRSF2*, *U2AF1*). These data were in line with clinical and biological characteristics of our cohort (older age, secondary or multilineage dysplasia patterns).^25^, ^26^ Mutations frequently identified in *de novo* AML of younger adults^2^ such as *FLT3*‐ITD and *NPM1* were rare (respectively, n = 3, 16% and n = 1, 5%). *ASXL1* mutations were the most frequent ones, and it should be noted that the majority of t(4;12) cases (n = 12/19, 63%) in our cohort fulfilled criteria for AML with MDS‐related changes (MRC‐AML) (multilineage dysplasia, MDS‐related cytogenetic abnormalities), which harboured frequent *ASXL1* mutations.^27^ The t(4;12) cases also shared with MRC‐AML frequent aberrant CD7 and CD56 expression.^28^

Interestingly, t(4;12) was the sole cytogenetic abnormality in nearly half of our cases, while when it was associated with additional cytogenetic abnormalities, these latter were in half cases present in an independent clone. We were also able to assess clonal architecture in 9 cases by combining cytogenetic and molecular data. We cannot exclude a potential selection bias due to cell culture affecting the chromosomal abnormalities frequencies. However, interphase FISH was used in 8 of 9 patients, limiting this effect. We observed that, contrary to what is classically observed for other recurrent chromosomal translocations, t(4;12) could be either an initiating or a secondary event in AML evolution. When t(4;12) was an initiating lesion, associated mutations were almost all in epigenetic regulators (*ASXL1*, *IDH2*, *TET2*) or splicing machinery (*SRSF2*). Moreover, no mutation in signalling pathway was observed, which contrasts with what is classically found with initiating chromosomal aberrations (eg del(20q), CBF or *MLL* translocations) and in cases in which t(4;12) was a secondary event. More studies would be necessary to understand whether different biological processes linked to t(4;12) and potential different breakpoints/translocation partners underlined these two different situations (initiating vs. secondary).

Definitive conclusions about clinical outcomes are difficult to determine due to the limited number of patients and the heterogeneity of therapeutic regimens used in our cohort. However, the presence of t(4;12) does not seem to confer chemoresistance as compared to results reported for those of equivalent age receiving induction chemotherapy^26^ or hypomethylating agents.^29^ Prognosis remains poor with a median PFS and OS of respectively 5 and 7.8 months but is in line with survival rates observed in AML patients of similar age^29^ and those harbouring similar cytogenetic/molecular characteristics.^26^

Here, we report the largest series of myeloid neoplasms with t(4;12). The t(4;12) cases harboured many characteristics of MRC‐AML (multilineage dysplasia, MDS‐related cytogenetic abnormalities, frequent *ASXL1* mutations) and a poor prognosis. Interestingly and contrary to what is classically observed with recurrent AML translocations, FISH and molecular analyses revealed that t(4;12) is not always a founding oncogenic event. Finally, t(4;12) does not seem to confer chemoresistance but its prognosis remains poor, which might be more closely linked to older age, other cytogenetic and molecular‐associated features rather than being specific.

## CONFLICT OF INTEREST

No relevant COI.

## AUTHOR CONTRIBUTION

**Vincent Parinet:** Conceptualization (equal); Data curation (equal); Formal analysis (lead); Validation (equal); Writing‐original draft (equal). **Elise Chapiro:** Conceptualization (equal); Data curation (equal); Formal analysis (equal); Investigation (equal); Methodology (equal); Supervision (equal); Validation (equal); Writing‐review & editing (equal). **Audrey Bidet:** Data curation (equal); Formal analysis (equal); Investigation (equal); Writing‐review & editing (equal). **Baptiste Gaillard:** Data curation (equal); Formal analysis (equal); Investigation (equal); Validation (equal); Writing‐review & editing (equal). **Odile Maarek:** Data curation (equal); Formal analysis (equal); Investigation (equal); Validation (equal); Writing‐review & editing (equal). **Laurence Simon:** Conceptualization (equal); Data curation (equal); Formal analysis (equal); Investigation (equal); Validation (equal); Writing‐review & editing (equal). **Christine Lefebvre:** Data curation (equal); Formal analysis (equal); Investigation (equal); Validation (equal); Writing‐review & editing (equal). **Sabine Defasque:** Data curation (equal); Formal analysis (equal); Investigation (equal); Validation (equal); Writing‐review & editing (equal). **Marie‐Joëlle Mozziconacci:** Data curation (equal); Formal analysis (equal); Methodology (equal); Validation (equal); Writing‐review & editing (equal). **Anne Quinquenel:** Data curation (equal); Formal analysis (equal); Methodology (equal); Validation (equal); Writing‐review & editing (equal). **Mathieu Decamp:** Data curation (equal); Formal analysis (equal); Methodology (equal); Validation (equal); Writing‐review & editing (equal). **François lifermann:** Data curation (equal); Formal analysis (equal); Methodology (equal); Validation (equal); Writing‐review & editing (equal). **Nadia Ali Ammar:** Data curation (equal); Formal analysis (equal); Methodology (equal); Writing‐review & editing (equal). **Agathe Maillon:** Data curation (equal); Formal analysis (equal); Investigation (equal); Validation (equal); Writing‐review & editing (equal). **Marine Baron:** Data curation (equal); Formal analysis (equal); Investigation (equal); Validation (equal); Writing‐review & editing (equal). **Melanie Martin:** Data curation (equal); Formal analysis (equal); Investigation (equal); Validation (equal); Writing‐review & editing (equal). **Stéphanie Struski:** Data curation (equal); Formal analysis (equal); Investigation (equal); Validation (equal); Writing‐review & editing (equal). **Dominique Penther:** Data curation (equal); Formal analysis (equal); Investigation (equal); Validation (equal); Writing‐review & editing (equal). **Jean Baptiste Micol:** Data curation (equal); Formal analysis (equal); Investigation (equal); Validation (equal); Writing‐review & editing (equal). **Nathalie Auger:** Data curation (equal); Formal analysis (equal); Investigation (equal); Validation (equal); Writing‐review & editing (equal). **Chrystele Bilhou‐Nabera:** Data curation (equal); Formal analysis (equal); Investigation (equal); Validation (equal); Writing‐review & editing (equal). **Sylvie Tondeur:** Data curation (equal); Formal analysis (equal); Investigation (equal); Validation (equal); Writing‐review & editing (equal). **Florence NGUYEN KHAC:** Data curation (equal); Formal analysis (equal); Investigation (equal); Validation (equal); Writing‐review & editing (equal). **Pierre Hirsch:** Conceptualization (equal); Data curation (equal); Formal analysis (equal); Investigation (equal); Methodology (lead); Supervision (equal); Validation (equal); Writing‐review & editing (equal). **Damien Roos‐Weil:** Conceptualization (lead); Formal analysis (lead); Funding acquisition (equal); Investigation (equal); Methodology (lead); Supervision (lead); Validation (lead); Writing‐original draft (lead); Writing‐review & editing (lead). **Jean‐Alain Martignoles:** Data curation (equal); Formal analysis (equal); Investigation (equal); Validation (equal); Writing‐review & editing (equal).

## Supporting information

Fig S1

Supplementary Material
