# Supplementary material for: Myeloid malignancies with translocation t(4;12)(q11‐13;p13): molecular landscape, clonal hierarchy and clinical outcomes
Source: J Cell Mol Med. 2021 Sep 7;25(20):9557–66. doi: 10.1111/jcmm.16895 (PMC8505829; doi:10.1111/jcmm.16895)
Supplement: Supplementary file 1 — Fig S1 [file JCMM-25-9557-s002.docx]

**Supplementary Figures**

**Supplementary Figure S1**


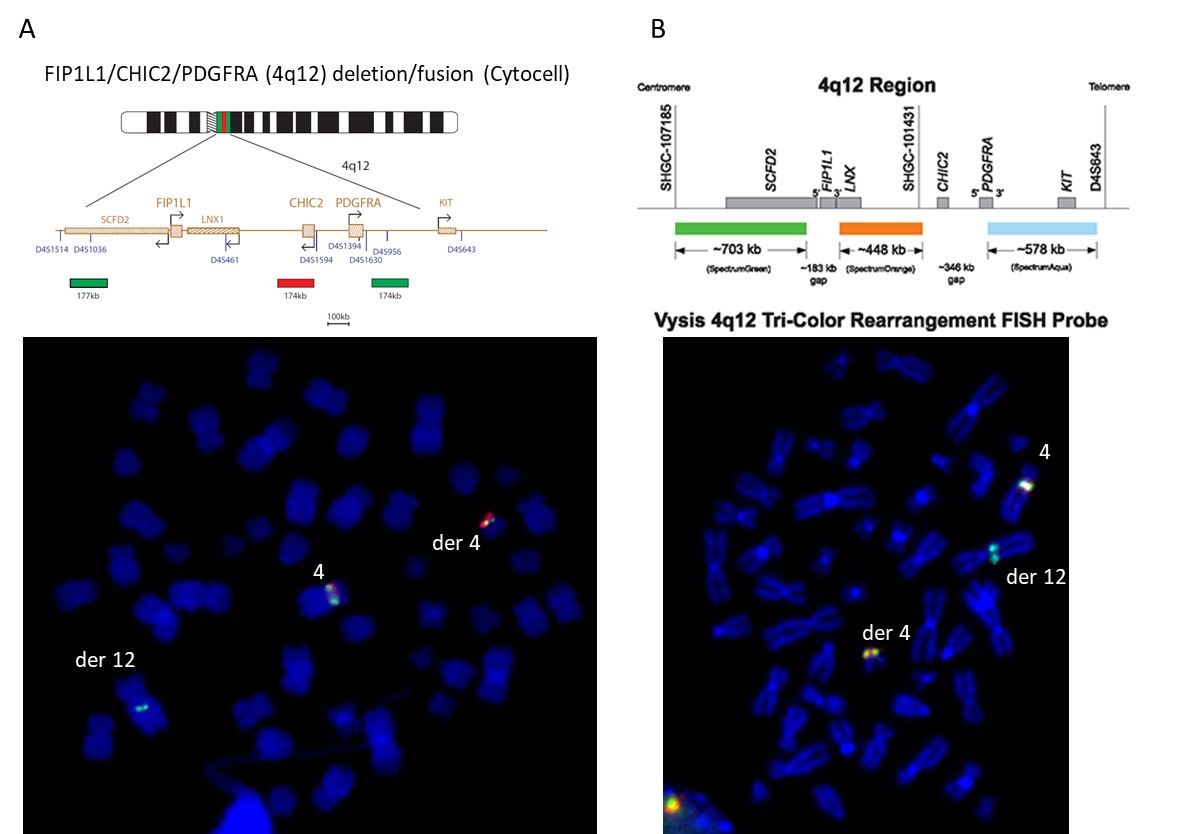


**Supplementary figures legends**

**Supplementary Figure S1.** Schematic representation of the 4q12 region covered by the two probes used in the study and representative metaphases for (A) Cytocell probe and (B) Vysis probe. The observed profiles indicate a breakpoint localized between the genes *CHIC2* and *PDGFRA*.
